# Sudden Infant Death Syndrome Mortality Trends and Socioeconomic Inequalities Worldwide: Evidence from the Global Burden of Disease Study

**DOI:** 10.3390/children12060747

**Published:** 2025-06-09

**Authors:** Ze Tang, Ziwei Wang, Xinbao Wang

**Affiliations:** Department of Pediatrics, Beijing Friendship Hospital, Capital Medical University, Beijing 100050, China

**Keywords:** sudden infant death syndrome, global burden of disease, mortality, disability-adjusted life years, sociodemographic index

## Abstract

**Background:** Sudden Infant Death Syndrome (SIDS) remains an important global health concern despite its decline in recent decades. This research assesses the global, regional, and national tendencies in SIDS mortality and DALYs from 1991 to 2021, highlighting the differences across various sociodemographic indexes (SDIs). **Methods**: Utilizing data from the Global Burden of Disease (GBD) study 2021, SIDS mortality and DALYs were evaluated across different global regions, SDI categories, and age groups. The trends over the study period were determined by conducting estimated annual percentage change (EAPC) analyses. **Results**: Between 1991 and 2021, the global SIDS mortality rate reduced greatly from 74,782 deaths (58.72 per 100,000 infants) to 30,608 deaths (24.16 per 100,000 infants), showing an EAPC of −3.01%. Similarly, the global DALYs decreased from 6,710,608 to 2,746,174. The biggest decline (EAPC: −5.25%) occurred in the high-SDI regions, whereas the low-SDI regions displayed a minimal decline (EAPC: −2.74%). Infants who were 1–5 months old uniformly had the highest mortality and DALY rates. Gender differences persisted, with larger rates discovered among males. The regional differences remained prominent, with the low-SDI states experiencing a much higher burden. **Conclusions**: Although there have been remarkable global advancements, great differences in the SIDS burden persist, mainly boosted by socioeconomic unfairness and healthcare access. Improved targeted interventions mitigating these modifiable risks and enhancing healthcare infrastructure in low-SDI regions are the keys to further reducing the global SIDS burden.

## 1. Introduction

Sudden Infant Death Syndrome (SIDS) is defined as the sudden death of an infant under one year of age that remains uninterpreted after a full examination, such as an autopsy, a death scene review, and a clinical history assessment [1]. First formally identified in the late 1960s, SIDS appeared as a unique diagnostic entity after the 1969 National Institute of Child Health and Human Development conference, which was designed to standardize the definitions for unexpected infant death [2]. The etiology of SIDS is multifactorial, involving an integration of genetic, environmental, and developmental elements [3,4]. This “triple-risk model” postulates that SIDS happens when an infant with internal vulnerability (e.g., brainstem abnormalities influencing cardiorespiratory control) is exposed to external stressors (e.g., prone sleeping) during a key growth period (typically 2-4 months of age) [5,6]. Genetic polymorphisms in serotonin and dopamine routes, including *5-HTT * and *DAT1*, have been implicated in changing autonomic regulation and the growing SIDS risk [7,8,9].  

SIDS remains the main cause of post-neonatal mortality in high-income states, with an incidence of 0.5–2.0 per 1000 live births [10]. Epidemiological research emphasizes that non-Hispanic Black, American Indian, and Alaskan Native populations have higher SIDS rates, likely because of socioeconomic unfairness and changes in secure sleep measures [11]. Prematurity, low birth weight, and respiratory infections (e.g., RSV) are common comorbidities linked to SIDS [12]. Maternal smoking during pregnancy increases the risk twofold, possibly due to nicotine’s effects on fetal neurodevelopment [13]. The complications extend beyond infant mortality, with families experiencing prolonged psychological trauma, including guilt and post-traumatic stress disorder [14]. Prone or side sleeping positions increase the SIDS risk from two- to thirteen-fold, as they impair airway patency and reduce arousal responsiveness [15]. Bed sharing, particularly with soft bedding or on couches, elevates this risk due to accidental suffocation or overheating [16,17]. The key characteristics identified include a higher prevalence in male infants; seasonality, with more cases reported in winter; and associations with prone sleeping positions and maternal smoking [15,18]. Park et al. reported a global decrease in SIDS mortality and disease burden based on the GBD 2019 data. However, these data are not comprehensive. Updates are available in GBD 2021.  

This article analyzes the mortality and DALYs of Sudden Infant Death Syndrome over the last 30 years using the GBD 2021 database. It examines global incidence and population and regional distribution, providing important information for clinical epidemiologists to implement the necessary preventive measures.  

## 2. Methods

### 2.1. Overview and Data Collection

The Global Burden of Disease (GBD) Database 2021 is an essential source of data that provides in-depth analyses of health outcomes worldwide (https://vizhub.healthdata.org/gbd-results/  accessed on 16 March 2025). Managed by the Institute for Health Metrics and Evaluation (IHME), it represents the most comprehensive collection of data regarding global health status, focusing on the impact of diseases, injuries, and risk factors across 204 countries and territories. The GBD database was built using an integrated approach, combining epidemiological data, vital statistics, health surveys, and other national datasets [19].  

This study identified the cause of death or injury because of SIDS and analyzed the mortality rate and related disability-adjusted life years (DALYs) among infants under one year old globally, regionally, and nationally by examining data from the GBD 2021 database. Linear regression in infants was employed to calculate estimated annual percentage changes (EAPCs).

### 2.2. Sociodemographic Index

As a composite measure, the Sociodemographic Index (SDI) is used to evaluate the growth level of a nation or region using three core indexes: total fertility rate under age 25 (or female total fertility rate under 25 years old); mean years of schooling for those aged 15 and older; and lag-distributed income per capita (lagged distribution of per capita income). It offers a basic understanding of the socioeconomic background of a region and assists in examining the association between growth advancement and public health results. The SDI varies from 0 to 1, with higher values suggesting greater socioeconomic progress. This research classified the states and regions into five SDI quintiles (low, low–medium, medium, medium–high, and high) to investigate the association between SIDS incidence and socioeconomic growth (Table S1 in Supplementary Materials) [20].

### 2.3. Disability-Adjusted Life Years

As a measure employed in public health research, DALYs quantify the overall burden of disease. They integrate the influence of premature mortality and the burden of morbidity, enabling the evaluation of the total years lost because of illness, disability, or early death. A single DALY stands for one lost year of “healthy” life. The calculation of DALYs offers an integrated measure of how much future life is lost and how much current life is spent in a less-than-fully healthy state [21].

### 2.4. SDI Analysis and Visualization

Statistical analysis of the relationship between SDI and SIDS death or DALYs rates was performed using Spearman’s rank correlation coefficient. All analyses were conducted using R software (version 4.4.2, R Foundation for Statistical Computing, Vienna, Austria) with the following packages: dplyr: for data cleaning, manipulation, and preprocessing, ggplot2: for data visualization and graphical representation. Statistical significance was set at *p* < 0.05, with 95% confidence intervals reported where applicable.

### 2.5. Cross-Country Inequality Analysis

We assessed cross-country inequalities in SIDS mortality using the Slope Index of Inequality (SII) and Concentration Index of Inequality (CII) [22,23]. Countries were ranked by SDI, reflecting income, education, and fertility. The SII was estimated via weighted regression of mortality on SDI rank to capture absolute inequality. The CII was calculated using Lorenz concentration curves to assess relative inequality, with 1000 bootstrap iterations used for confidence intervals. Negative SII and CII values indicate higher mortality in lower-SDI countries. The data points are displayed with varying sizes to represent the population scale. The size of each point is proportional to the population size of the corresponding country or region, where larger points indicate countries with larger populations and smaller points represent countries with smaller populations. These metrics quantify disparities across the development levels and were computed using R software.

### 2.6. Statistical Analysis

The mortality and DALYs rates were analyzed to determine the burden of SIDS. This burden was measured per 100,000 individuals with a 95% uncertainty interval using the Global Burden of Disease algorithm [24]. The trends were evaluated by calculating EAPC. A negative upper limit of the EAPC and its 95% confidence interval indicates a decline, while a positive lower limit suggests a rise. Global maps were created using R software with specialized packages for geospatial data processing and visualization, including sf for spatial data processing and geographic information systems functionality, magrittr for pipeline operations and data manipulation workflows using the pipe operator, grDevices for color palette generation using the color Ramp Palette function (built-in R package), and ggplot2 for base plotting and map visualization, along with additional geospatial packages as required for coordinate reference systems and map projections [25,26]. Statistical analyses were conducted using R software(version 4.4.2), and significance was set at *p* < 0.05.

## 3. Results

### 3.1. Global SIDS Trends

#### 3.1.1. Mortality

From 1991 to 2021, the global burden of SIDS decreased significantly (Table 1). Globally, deaths attributed to SIDS dropped from 74,782.08 (95% UI: 44,795.92–114,047.11) in 1991 to 30,607.69 (95% UI: 17,810.17–41,094.37) in 2021, with an estimated annual percentage change (EAPC) rate of −3.07% (95% CI: −3.23 to −2.90). Initially, in 1991, the global mortality rate for SIDS was 58.72 per 100,000 (95% UI: 35.17–89.55), showing a notable decline to 24.16 per 100,000 (14.06–32.44).

**Figure 1:**
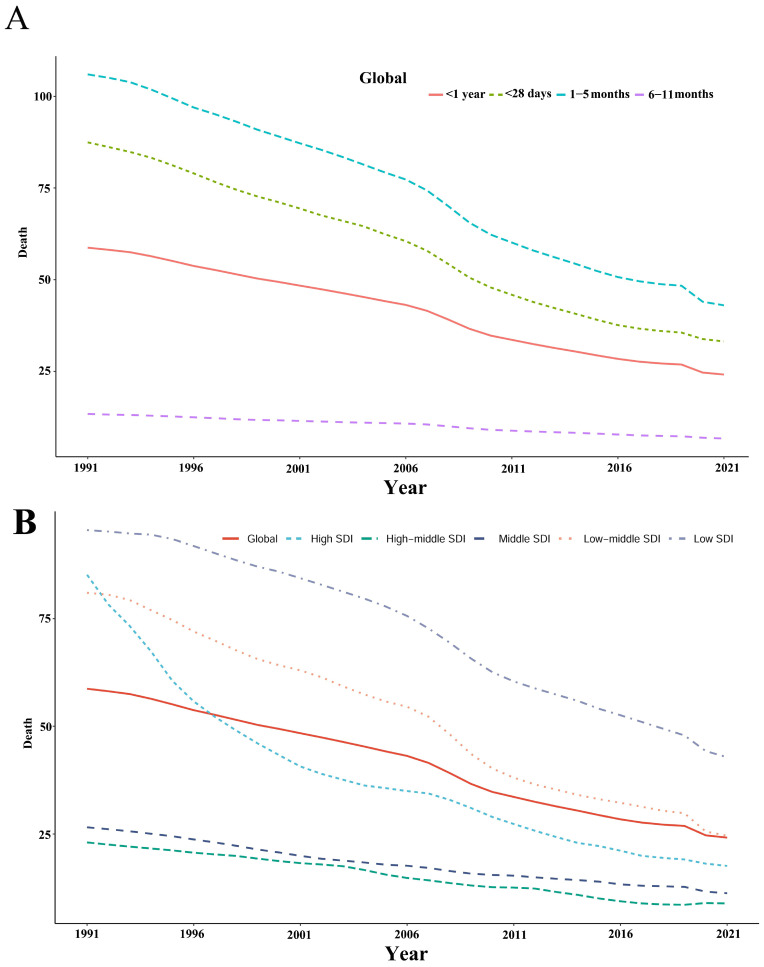
(**A**) Significant decrease in SIDS death rates among infants of different ages from 1991 to 2021. (**B**) Significant decrease in SIDS death rates across five Sociodemographic Index (SDI) categories from 1991 to 2021.

The SIDS death rate significantly decreased across all infant age groups (Figure 1A). The highest death rates consistently occurred among infants aged 1–5 months, declining from 105.98 per 100,000 (95% UI: 64.61–161.19) in 1991 to 43.01 per 100,000 (95% UI: 25.24–57.48) in 2021. Similarly, the death rates for infants younger than 28 days dropped from 87.45 per 100,000 (95% UI: 42.50–143.98) in 1991 to 33.16 per 100,000 (95% UI: 18.23–47.95) by 2021. Infants aged 6–11 months had the lowest rates, reducing from 13.42 per 100,000 (95% UI: 7.46–20.15) in 1991 to 6.74 per 100,000 (95% UI: 2.98–11.69) in 2021. Overall, the global death rate for infants younger than one year declined notably from 58.72 per 100,000 (95% UI: 35.17–89.55) in 1991 to 24.16 per 100,000 (95% UI: 14.06–32.44) by 2021.  

#### 3.1.2. DALYs

From 1991 to 2021, the global DALYs declined from 6,710,608.37 (95% UI: 4,019,964.49–10,234,783.01) in 1991 to 2,746,174.49 (95% UI: 1,598,180.42–3,686,289.79) in 2021 (EAPC: −3.07%; 95% CI: −3.23 to −2.90). Initially, in 1991, the global DALY rate for SIDS was 5269.18 per 100,000 (95% UI: 3156.48–8036.37), showing a notable decline to 2167.56 per 100,000 (95% UI: 1261.44–2909.59) by 2021 (Table 1).

From 1991 to 2021, DALYs due to SIDS significantly decreased across all infant age groups (Figure S1 in Supplementary Materials). Infants aged 1–5 months consistently showed the highest DALY rates, decreasing markedly from 9513.14 per 100,000 (95% UI: 5799.31–14,468.59) in 1991 to 3860.26 per 100,000 (95% UI: 2265.47–5159.50) in 2021. Similarly, for infants younger than 28 days, the DALYs decreased from 7868.07 per 100,000 (95% UI: 3824.30–12,954.23) in 1991 to 2983.96 per 100,000 (95% UI: 1640.48–4314.04) in 2021. The lowest rates were reported among infants aged 6–11 months, declining from 1198.61 per 100,000 (95% UI: 666.07–1799.92) in 1991 to 601.69 per 100,000 (95% UI: 266.35–1044.59) in 2021. Overall, for infants aged younger than one year, the global DALY rate decreased substantially from 5269.18 per 100,000 (95% UI: 3156.48–8036.37) to 2167.56 per 100,000 (95% UI: 1261.44–2909.59) over the three decades studied.

### 3.2. SIDS in SDI Trends

#### 3.2.1. Mortality

This study reveals a significant inverse correlation between SDI and SIDS mortality rates across 21 global regions from 1991 to 2021 (Spearman’s r = −0.3854, 95% CI: −0.4521–0.3106, *p* < 0.001), indicating higher SIDS burden in socioeconomically disadvantaged areas. (Figure 1B). In 1991, the highest mortality rate was observed in the low-SDI regions at 95.53 per 100,000 (95% UI: 46.06–152.74), which fell to 42.80 per 100,000 (95% UI: 22.28–62.67) by 2021. The high-SDI regions experienced a significant drop from 85.20 per 100,000 (95% UI: 82.39–88.15) in 1991 to 17.60 per 100,000 (95% UI: 15.36–19.92) in 2021. The middle-SDI regions saw reductions from 26.56 per 100,000 (95% UI: 15.38–37.36) in 1991 to 11.26 per 100,000 (95% UI: 7.00–15.58) in 2021. Likewise, the low–middle-SDI regions decreased from 81.01 per 100,000 (95% UI: 39.21–145.14) in 1991 to 24.60 per 100,000 (95% UI: 13.51–36.36) in 2021. The high–middle-SDI regions, which recorded the lowest rates, dropped from 23.04 per 100,000 (95% UI: 16.77–32.15) in 1991 to 8.89 per 100,000 (95% UI: 6.27–11.52) in 2021.

#### 3.2.2. DALYs

The Spearman correlation analysis between the DALYs rate of SIDS and SDI across 21 global regions (1991–2021) revealed a significant negative association (r = −0.3854, 95% CI: −0.4589 to −0.3160, *p*< 0.001) (Figure S2 in Supplementary Materials). All the SDI quintiles experienced declining trends, with the high-SDI regions showing the largest reduction from 7644.56 (95% UI: 7393.07–7909.22) in 1991 to 1578.99 (95% UI: 1378.06–1787.21) in 2021 (EAPC: −4.87%, 95% CI: from −5.10 to −4.63). The low-SDI regions had the highest burden in 1991 at 8572.14 (95% UI: 4133.70–13,705.30) but decreased to 3840.09 (95% UI: 1998.76–5622.32) in 2021, remaining the highest across all the SDI groups (EAPC: −2.74%, 95% CI: from −2.94 to −2.54). The DALY rate in the low–middle-SDI regions fell from 7270.27 (95% UI: 3518.31–13,026.42) to 2207.37 (95% UI: 1212.16–3262.24), while middle the SDI and high–middle-SDI regions showed notable reductions to 1010.71 (95% UI: 627.98–1398.25) and 797.57 (95% UI: 562.44–1033.51), respectively, in 2021.

### 3.3. Regional SIDS Trends

#### 3.3.1. Mortality

**Figure 2:**
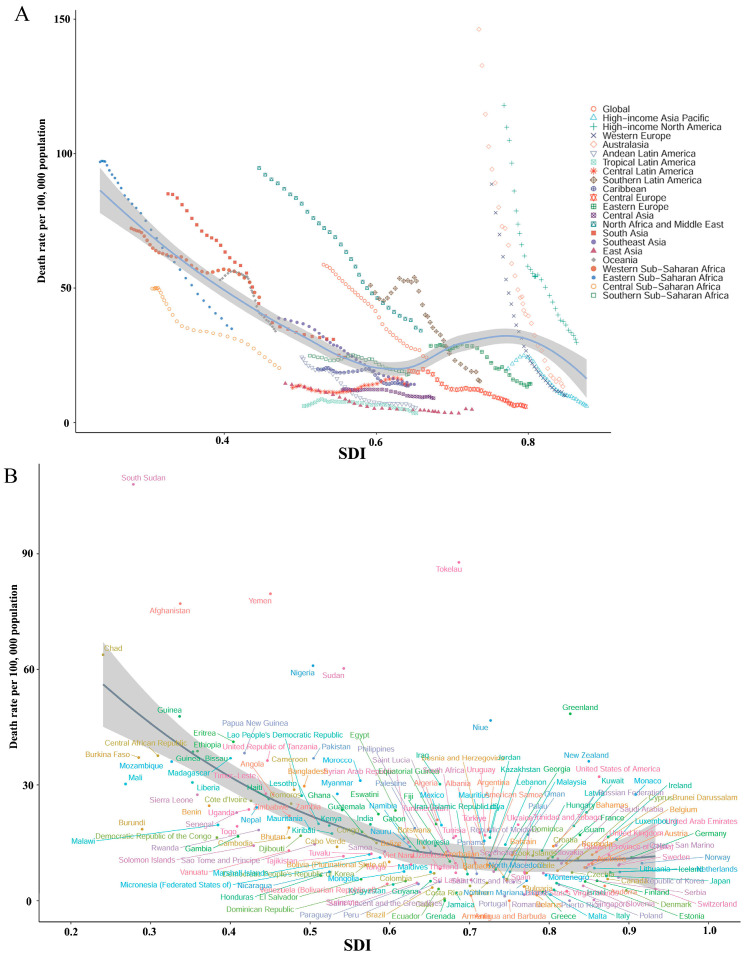
(**A**) Regional variation in SIDS death rates based on SDI categories from 1991 to 2021. (**B**) Global variation in SIDS death rates among infants in different countries based on SDI in 2021.

The SIDS mortality rates of the 21 regions were different between 1991 and 2021 (Figure 2A, Table 1). The high-income regions, including Australasia, showed the highest reduction in mortality rates, declining from 146.26 (95% UI: 136.30–156.86) to 13.37 (95% UI: 9.97–17.35) per 100,000. Similarly, North America, a high-income region, demonstrated substantial progress, with this value decreasing from 117.88 per 100,000 (95% UI: 113.35–122.80) in 1991 to 29.72 (95% UI: 25.19–34.22) in 2021. In contrast, the regions with lower SDI scores, such as eastern Sub-Saharan Africa, showed a less pronounced decline, from 96.91 deaths per 100,000 (95% UI: 47.65–157.32) to 34.91 deaths per 100,000 (95% UI: 18.79–51.65), indicating persistent challenges despite improvements. Notably, Central Latin America was the only region to report an increase, albeit minimal, in mortality rate from 12.99 (95% UI: 11.31–15.21) to 13.99 (95% UI: 10.29–19.06) per 100,000. SDI-related stratification revealed a strong inverse association between the SDI and SIDS mortality, with the higher-SDI regions achieving greater reductions.

**Figure 3:**
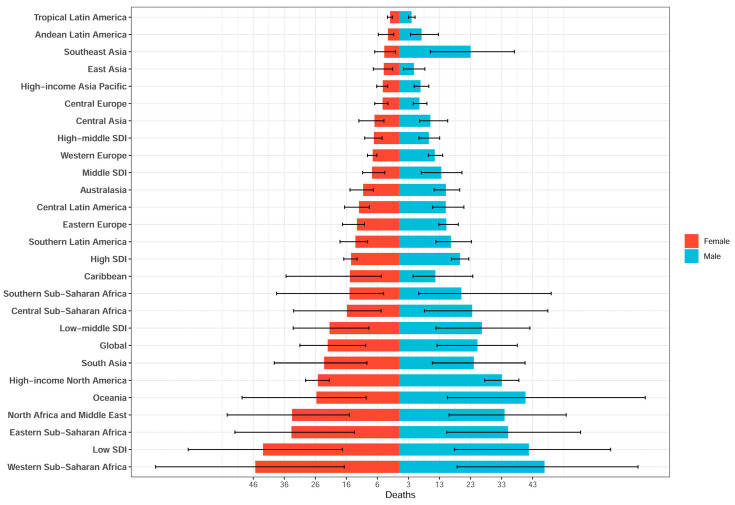
Noticeable disparities in SIDS death rates based on sex and region.

In 2021, notable disparities in SIDS mortality rates existed among the sexes (Figure 3). Globally, the male mortality rate (25.31 per 100,000; 95% UI: 12.28–38.16) exceeded that of females (22.93 per 100,000; 95% UI: 10.66–31.90). Elevated rates occurred in western (males: 46.92; 95% UI: 18.75–77.05) and eastern Sub-Saharan Africa (males: 35.19; 95% UI: 15.42–58.53), with similar trends among the female population. The high-income region of North America also showed higher rates among males (33.16; 95% UI: 27.63–38.64) versus females (26.11; 95% UI: 22.42–30.08). The lowest rates appeared in East Asia and the high-income Asia–Pacific regions for both sexes, reflecting substantial regional variation in SIDS mortality.

#### 3.3.2. DALYs

Between 1991 and 2021, the global burden of SIDS in children under 1 year, measured in DALYs, exhibited considerable variability in the 21 regions (Figure S3 in Supplementary Materials). The greatest reduction occurred in Australasia, where the DALY rate fell from 13,122.08 (95% UI: 12,228.55–14,073.20) in 1991 to 1200.04 per 100,000 (95% UI: 894.48–1557.24) in 2021. The high-income regions of North America and Western Europe also showed marked decreases from 10,578.36 (10,171.88–11,019.53) to 2666.52 (2260.16–3071.11) and from 7958.31 (7598.55–8332.61) to 900.90 (755.14–1074.23), respectively. The value for Eastern Europe declined from 2555.92 (2167.81–3044.49) to 1295.62 (1080.08–1651.70). However, several low-SDI regions had more modest improvements. In eastern Sub-Saharan Africa, the DALY rate changed from 8696.03 (4275.79–14,118.58) to 3132.38 (1686.15–4633.44), while those for North Africa and the Middle East and western Sub-Saharan Africa also remained relatively high at 3070.16 (1759.92–4617.29) and 4179.95 (2046.05–46,299.52), respectively, in 2021.

In 2021, the DALYs due to SIDS varied among the sexes (Figure S4 in Supplementary Materials). Globally, males had slightly higher DALYs (2270.69 per 100,000; 95% UI: 1102.33–3424.11) than females (2057.31; 95% UI: 956.74–2861.75). The highest rates appeared in western Sub-Saharan Africa (males: 4208.95; females: 4149.80 per 100,000). Elevated rates (>3100 per 100,000) also occurred in eastern Sub-Saharan Africa and South Asia. Conversely, tropical Latin America and East Asia had notably lower DALYs (e.g., tropical Latin American males: 364.26; females: 256.94). Western Europe (males: 1036.93; females: 757.81) and the high-income region Asia–Pacific similarly reported lower rates.

### 3.4. National SIDS Trends

#### 3.4.1. Mortality

**Figure 4:**
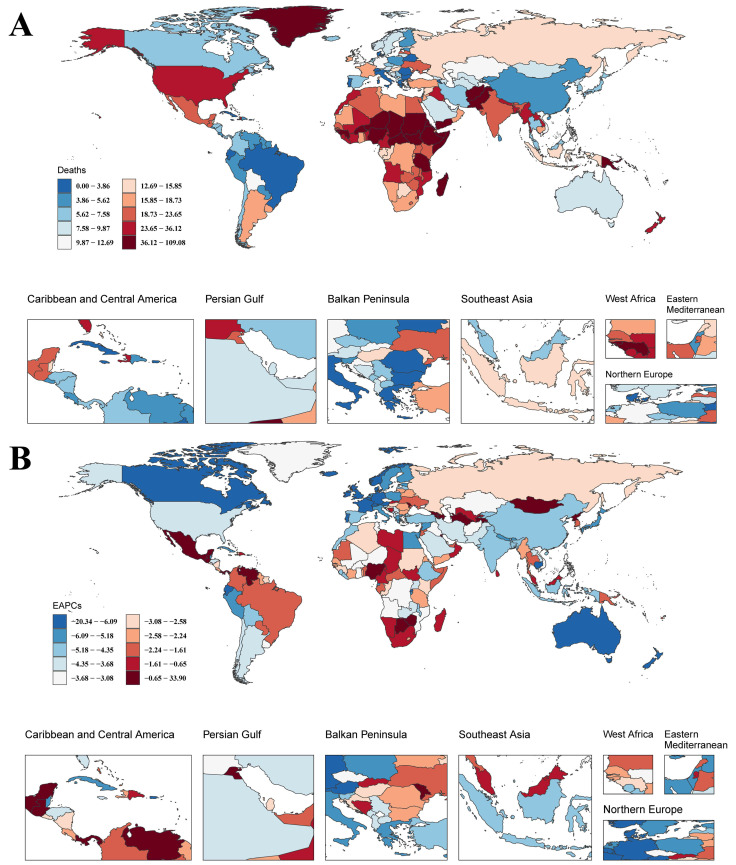
(**A**) National variation in SIDS death rates in 2021. (**B**) National EAPC variation in SIDS death rates from 1991 to 2021.

This study analyzed the correlation between SIDS mortality and the SDI across 204 countries/territories in 2021 using the GBD database. A significant negative correlation was observed (Spearman’s r = −0.5672, 95% CI: −0.6596 to −0.4419, *p* < 0.001) (Figure 2B and Figure 4A). The low-SDI countries, such as South Sudan, reported the highest SIDS mortality rate at 108.00 per 100,000 infants (95% UI: 50.15–199.07), followed by Tokelau (87.78 per 100,000; 95% UI: 38.64–160.42) and Yemen (79.69 per 100,000; 95% UI: 35.82–160.61). Other countries had notably high rates, including Afghanistan (77.12 per 100,000), Chad (63.80 per 100,000), and Nigeria (60.97 per 100,000). Conversely, Antigua and Barbuda exhibited the lowest rate at 0.00017 per 100,000 (95% UI: 0.00012–0.00024), with Puerto Rico (0.00254 per 100,000; 95% UI: 0.00174–0.00352) and Grenada (0.0246 per 100,000; 95% UI: 0.0157–0.0361) also reporting minimal rates. The high-SDI regions, such as the United States of America and New Zealand, had intermediate SIDS mortality rates of 32.12 (95% UI: 27.26–37.07) and 36.12 (95% UI: 27.99–45.02), respectively. The reductions were most notable in the middle-to-high-SDI countries, illustrating improvements in health correlated with increased socioeconomic development.

#### 3.4.2. DALYs

This study investigated the association between SDI and SIDS DALYs rates across 204 countries/territories using 2021 Global Burden of Disease data. Spearman correlation revealed a significant inverse relationship (r = −0.5671, 95% CI: −0.6594 to −0.4416, *p* < 0.001) (Figures S5 and S6 in Supplementary Materials). South Sudan exhibited the highest DALY rate at 9689.88 per 100,000 infants (95% UI: 4499.77–17,860.46), followed by Tokelau (7874.84 per 100,000; 95% UI: 3465.23–14,391.48) and Yemen (7148.93 per 100,000; 95% UI: 3212.54–14,413.56). Some other countries with considerable DALY rates included Afghanistan (6919.54 per 100,000), Chad (5723.04 per 100,000), and Nigeria (5468.91 per 100,000). Conversely, countries like Antigua and Barbuda (0.016 per 100,000; 95% UI: 0.011–0.022), Puerto Rico (0.23 per 100,000; 95% UI: 0.16–0.32), and Grenada (2.21 per 100,000; 95% UI: 1.41–3.24) showed a significantly lower burden. The high-SDI regions, such as the United States, recorded a moderate DALY rate of 2882.36 per 100,000 infants (95% UI: 2445.95–3326.49), while New Zealand reported 3241.22 per 100,000 infants (95% UI: 2511.64–4039.71). These declines were most prominent in the middle-to-high-SDI regions, emphasizing enhanced health progress associated with socioeconomic development.

### 3.5. EAPC in Death and DALY Rates by Region

From 1991 to 2021, the global burden of SIDS showed a decreasing trend in most regions, with statistically significant declines in the death rates observed globally and across nearly all the SDI and GBD regions (Table 1). Globally, the EAPC in death rate was −3.07% (95% CI: −3.23 to −2.90). The largest decreases were reported in Australasia [−7.38% (95% CI: from −7.67 to −7.08)], Western Europe [−6.73% (−7.13 to −6.32)], and Andean Latin America [−5.25% (−5.65 to −4.84)]. The high-SDI regions also experienced substantial declines, such as the high-income regions Asia–Pacific [−4.68% (−5.04 to −4.31)] and North America [−4.08% (−4.37 to −3.79)]. In contrast, Central Latin America was the only region to exhibit a statistically significant increase, with an EAPC of 0.94% (95% CI: 0.54 to 1.34), indicating a rising trend in SIDS mortality. All the other regions demonstrated negative EAPCs, with the 95% CIs not crossing zero.

From 1991 to 2021, most countries experienced a decline in the death rate of SIDS, though disparities persisted (Figure 4B). Canada [EAPC: −8.24%, 95% CI: −8.85 to −7.62], Belgium [−8.06%, 95% CI: −8.42 to −7.70], and Australia [−7.91%, 95% CI: −8.40 to −7.41] showed the steepest declines. Other high-income nations—including Germany [−7.73%, 95% CI: −7.90 to −7.57], Switzerland [−7.61%, 95% CI: −8.00 to −7.21], and the U.K. [−6.53%, 95% CI: −6.85 to −6.21]—also reported significant reductions. In contrast, several countries had statistically significant increases, notably Georgia [33.56%, 95% CI: 26.59 to 40.91], Panama [4.09%, 95% CI: 3.70 to 4.48], and Mexico [2.34%, 95% CI: 1.88 to 2.80].

### 3.6. Inequalities in SIDS Mortality Rate

**Figure 5:**
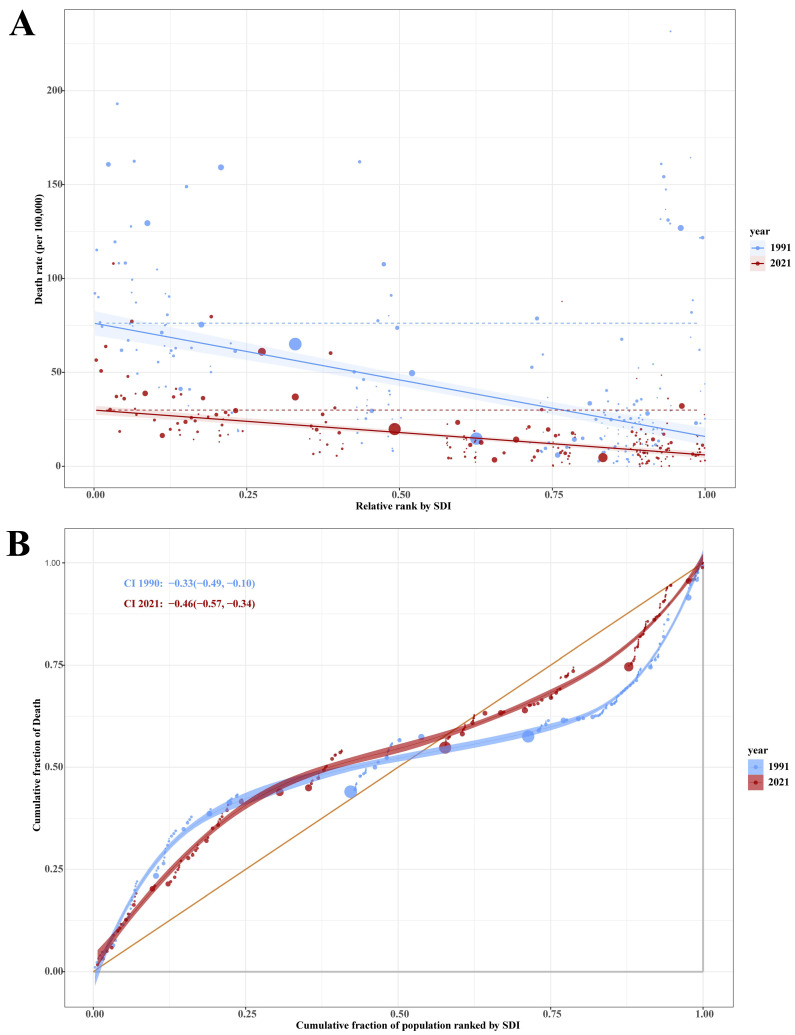
(**A**) Slope Index of Inequality (SII) disparities in Sudden Infant Death Syndrome (SIDS) mortality rates across countries stratified by Socio-demographic Index (SDI) from 1991 to 2021. (**B**) Cumulative distribution of SIDS deaths across populations ranked by SDI from 1991 to 2021. Blue markers represent 1991 data; red markers represent 2021 data. Each data point corresponds to an individual country, with marker size proportional to population size, larger markers indicate countries with greater populations.

Between 1991 and 2021, marked disparities were observed in the mortality rates of SIDS across the countries stratified by the SDI (Figure 5A,B). In 1991, the SII for SIDS mortality was −60.68 (95% CI: −70.62 to −50.73), indicating a steep absolute inequality in death rates across the SDI gradient. By 2021, the SII had declined to −23.90 (95% CI: −27.46 to −20.33), reflecting an improvement in absolute equity. The CII similarly reflected a persistent but worsening concentration of SIDS mortality in the lower-SDI regions over the study period. In 1991, the index was −0.33 (95% CI: −0.49 to −0.10), denoting that SIDS deaths were disproportionately concentrated among socioeconomically disadvantaged populations. By 2021, this inequality had intensified, with a CII of −0.46 (95% CI: −0.57 to −0.34).

## 4. Discussion

Globally, mortality due to SIDS has significantly declined over the past three decades, falling from 74,782 deaths in 1991 to 30,608 in 2021, with an estimated annual percentage change (EAPC) of −3.01% (95% CI: −3.18 to −2.84). This decline was uniform across all infant age groups, most remarkably among infants aged 1–5 months old (from 105.98 to 43.01 per 100,000). Similarly, the global DALYs related to SIDS reduced considerably, showing improved healthcare interventions and preventive practices across the world.

Marked regional differences persist. The dramatic reductions in SIDS mortality rates observed in high-income North America, Australia, and Western Europe during the 1990s can be attributed to the coordinated implementation of comprehensive prevention strategies that created threshold effects at specific SDI levels. The “Back to Sleep” campaigns launched in these regions during the early 1990s marked a pivotal intervention point, promoting supine sleep positioning and rapidly transforming parental behavior through widespread public health messaging [27,28]. These campaigns were accompanied by enhanced parental education programs that addressed multiple risk factors simultaneously, including safe sleep environments, breastfeeding promotion, and smoke exposure reduction [29,30]. The convergence of robust healthcare systems in these high-income regions facilitated the rapid adoption and dissemination of evidence-based practices, while standardized SIDS investigation protocols improved case identification and monitoring [31,32]. The steep mortality declines observed in Figure 2A reflect threshold effects where multiple protective factors, socioeconomic stability, healthcare access, public health infrastructure, and cultural receptivity to medical recommendations, converged at critical SDI levels, creating synergistic benefits that exceeded the sum of individual interventions [29]. By contrast, areas with a lower socioeconomic status, including eastern Sub-Saharan Africa and North Africa, showed less obvious reductions, highlighting socioeconomic determinants such as healthcare accessibility, education, and income level as the key elements influencing these SIDS results.

Socioeconomic inequalities remain a factor that greatly affects the incidence and mortality rates of SIDS. Low- and low–middle-SDI countries such as South Sudan, Yemen, and Afghanistan exhibited substantially higher mortality rates and DALYs compared to the high-SDI regions, highlighting persistent gaps in healthcare access, awareness, and infrastructure; these results are consistent with those reported in previous studies [33,34]. These disparities underscore the necessity for targeted interventions addressing education, economic stability, and healthcare delivery specifically tailored to vulnerable populations [35].

From 1991 to 2021, most countries experienced significant declines in SIDS mortality, particularly the high-income regions. However, a few countries, such as Georgia and Panama, showed rising trends. These disparities highlight uneven progress and emphasize the need for targeted interventions and strengthened health systems to reduce preventable infant deaths globally and address the emerging inequalities in the SIDS burden. A cross-country inequality analysis showed great differences in SIDS mortality across the various levels of socioeconomic growth. Although there has been an overall decline in the global mortality rate, both the Slope Index and the Concentration Index suggested persistent and significant pro-disadvantaged unfairness. SIDS mortality remained disproportionately concentrated in the low-SDI states, highlighting the ongoing burden in settings with restricted resources. Although absolute inequality (SII) reduced as time passed, relative inequality (CII) intensified, indicating uneven advancement. These outcomes emphasize the pressing demand for targeted public health interventions, enhanced health systems, and fair resource distribution to decrease preventable infant deaths and solve structural differences in child healthcare globally.

The risk factors for SIDS have been widely researched and classified into modifiable and non-modifiable elements. The modifiable risk elements encompass unsafe sleep environments, maternal smoking, overheating, and improper prenatal care, whereas the non-modifiable risk elements include genetic vulnerabilities, age, and gender [36,37,38]. Public health campaigns alerting people to modifiable risks, including secure sleep initiatives and anti-smoking contributions, have been useful, especially in developed regions, encouraging intensified focus and resource distribution in less-developed regions to maximize their influence [39,40].

To usefully address the inequalities discovered in SIDS mortality, public health policies must prioritize the fair allocation of resources, especially in prenatal and neonatal healthcare services. These policy suggestions include extending educational initiatives for caregivers, guaranteeing accessible healthcare for mother and infant populations, and combining systematic screening for known SIDS risk elements with routine healthcare measures [41,42]. In addition, improving healthcare infrastructure in underserved areas will decrease the global SIDS mortality rate and solve the socioeconomic problems that perpetuate these differences.

The limitations of this study encompass potential differences in data quality and reporting standards across regions, which may have affected the precision of regional comparisons. Moreover, not having individual-level data restricted our capacity to provide a detailed discussion on specific risk elements, such as genetic predispositions and accurate socio-environmental influences.

Future studies should prioritize improving global data standardization and gathering detailed individual-level epidemiological, genetic, and socioeconomic information to allow for precise interventions. In addition, future studies should investigate the influence of targeted interventions in low-SDI regions, assess genetic and metabolic screening programs, and promote cross-regional cooperation to further reduce SIDS mortality. Moreover, public health policies must pay attention to fair healthcare access, sustained parental education campaigns, and culturally appropriate interventions that are personalized to the regional contexts.

To sum up, this research highlights the great global advancement in decreasing SIDS mortality rates and DALYs over the past three decades while emphasizing persistent regional differences, which demand focused, multi-dimensional intervention measures. Solving these problems will be the key to decreasing the global burden of SIDS.

## Figures and Tables

**Table 1:** Mortality and disability-adjusted life years from Sudden Infant Death Syndrome (SIDS) between 1991 and 2021.

	Death					DALYs				
location	1991 Cases	1991 Rate (95% UI, per 100,000)	2021 Cases	2021 Rate (95% UI, per 100,000)	EAPC (95% CI, %)	1991 Cases	1991 Rate (95% UI, per 100,000)	2021 Cases	2021 Rate (95% UI, per 100,000)	EAPC (95% CI, %)
Global	74,782.08 (44,795.92, 114,047.11)	58.72 (35.17, 89.55)	30,607.69 (17,810.17, 41,094.37)	24.16 (14.06, 32.44)	−3.07 (−3.23, −2.90)	6,710,608.37 (4,019,964.49, 10,234,783.01)	5269.18 (3156.48, 8036.37)	2,746,174.49 (1,598,180.42, 3,686,289.79)	2167.56 (1261.44, 2909.59)	−3.07 (−3.23, −2.90)
High SDI	10,453.91 (10,110.10, 10,815.84)	85.20 (82.39, 88.15)	1805.28 (1575.55, 2043.32)	17.60 (15.36, 19.92)	−4.87 (−5.10, −4.63)	938,016.55 (907,158.05, 970,491.28)	7644.56 (7393.07, 7909.22)	162,001.17 (141,386.97, 183,364.52)	1578.99 (1378.06, 1787.21)	−4.87 (−5.10, −4.63)
High–middle SDI	4023.49 (2928.58, 5613.33)	23.04 (16.77, 32.15)	1057.92 (746.05, 1370.97)	8.89 (6.27, 11.52)	−3.55 (−3.75, −3.36)	952,529.74 (551,494.75, 1,340,306.29)	2383.00 (1379.71, 3353.12)	322,450.57 (200,345.06, 446,089.81)	1010.71 (627.98, 1398.25)	−3.55 (−3.75, −3.36)
Middle SDI	10,614.64 (6146.03, 14,933.85)	26.56 (15.38, 37.36)	3593.20 (2232.31, 4970.37)	11.26 (7.00, 15.58)	−2.77 (−2.84, −2.70)	2,667,421.87 (1,290,850.09, 4,779,320.64)	7270.27 (3518.31, 13,026.42)	838,441.51 (460,421.73, 1,239,117.82)	2207.37 (1212.16, 3262.24)	−2.77 (−2.84, −2.70)
Low–middle SDI	29,723.64 (14,385.74, 53,251.45)	81.01 (39.21, 145.14)	9344.65 (5130.85, 13,811.88)	24.60 (13.51, 36.36)	−3.99 (−4.22, −3.76)	1,788,044.64 (862,240.69, 2,858,761.74)	8572.14 (4133.70, 13,705.30)	1,326,431.33 (690,406.38, 1,942,045.37)	3840.09 (1998.76, 5622.32)	−3.99 (−4.22, −3.76)
Low SDI	19,926.22 (9607.89, 31,860.12)	95.53 (46.06, 152.74)	14,785.39 (7696.71, 21,646.02)	42.80 (22.28, 62.67)	−2.74 (−2.94, −2.54)	24,791.22 (11,961.79, 44,290.61)	2183.08 (1053.34, 3900.17)	5991.85 (3425.56, 9774.79)	490.21 (280.26, 799.71)	−2.74 (−2.94, −2.54)
Andean Latin America	276.22 (133.29, 493.42)	24.32 (11.74, 43.45)	66.76 (38.16, 108.90)	5.46 (3.12, 8.91)	−5.25 (−5.65, −4.84)	41,243.87 (38,435.56, 44,233.50)	13,122.03 (12,228.55, 14,073.20)	4245.98 (3164.88, 5509.85)	1200.04 (894.49, 1557.25)	−5.25 (−5.65, −4.84)
Australasia	459.70 (428.39, 493.03)	146.26 (136.30, 156.86)	47.32 (35.27, 61.40)	13.37 (9.97, 17.35)	−7.38 (−7.67, −7.08)	15,389.61 (8203.75, 30,602.90)	1783.08 (950.51, 3545.74)	9537.28 (4204.69, 18,184.54)	1230.63 (542.55, 2346.42)	−7.38 (−7.67, −7.08)
Caribbean	171.48 (91.40, 340.98)	19.87 (10.59, 39.51)	106.27 (46.86, 202.60)	13.71 (6.05, 26.14)	−1.46 (−1.71, −1.21)	20,430.38 (8014.75, 30,728.96)	1082.46 (424.65, 1628.11)	16,414.04 (10,492.25, 25,539.37)	812.19 (519.17, 1263.72)	−1.46 (−1.71, −1.21)
Central Asia	227.80 (89.35, 342.64)	12.07 (4.73, 18.15)	182.94 (116.91, 284.68)	9.05 (5.79, 14.09)	−1.15 (−1.35, −0.95)	28,366.40 (22,960.11, 34,463.07)	1717.93 (1390.51, 2087.15)	5615.67 (3899.74, 7933.26)	533.00 (370.14, 752.98)	−1.15 (−1.35, −0.95)
Central Europe	316.22 (255.95, 384.19)	19.15 (15.50, 23.27)	62.57 (43.45, 88.40)	5.94 (4.12, 8.39)	−4.04 (−4.31, −3.76)	56,792.10 (49,446.34, 66,512.49)	1165.66 (1014.88, 1365.17)	48,620.12 (35,751.39, 66,227.66)	1255.30 (923.05, 1709.90)	−4.04 (−4.31, −3.76)
Central Latin America	632.89 (551.04, 741.16)	12.99 (11.31, 15.21)	541.88 (398.38, 738.33)	13.99 (10.29, 19.06)	0.94 (0.54,1.34)	109,011.79 (38,501.53, 231,116.28)	4470.74 (1579.01, 9478.44)	77,962.31 (33,277.69, 148,928.45)	1815.08 (774.75, 3467.28)	0.94 (0.54,1.34)
Central Sub-Saharan Africa	1214.94 (428.94, 2576.72)	49.83 (17.59, 105.68)	869.00 (370.82, 1660.72)	20.23 (8.63, 38.66)	−2.95 (−3.19, −2.72)	293,987.19 (161,738.97, 461,697.41)	1302.36 (716.50, 2045.31)	51,899.15 (22,550.07, 86,663.58)	434.58 (188.83, 725.69)	−2.95 (−3.19, −2.72)
East Asia	3277.31 (1803.33, 5148.06)	14.52 (7.99, 22.81)	578.41 (251.30, 965.93)	4.84 (2.10, 8.09)	−4.75 (−5.40, −4.09)	71,681.17 (60,796.53, 85,382.95)	2555.92 (2167.81, 3044.49)	23,538.09 (19,622.19, 30,007.10)	1295.62 (1080.08, 1651.70)	−4.75 (−5.40, −4.09)
Eastern Europe	799.08 (677.74, 951.89)	28.49 (24.17, 33.94)	262.30 (218.67, 334.37)	14.44 (12.04, 18.40)	−2.71 (−3.19, −2.24)	735,818.53 (361,798.52, 1,194,649.51)	8696.03 (4275.80, 14,118.58)	413,069.04 (222,352.85, 611,013.28)	3132.39 (1686.15, 4633.44)	−2.71 (−3.19, −2.24)
Eastern Sub-Saharan Africa	8200.29 (4032.25, 13,311.42)	96.91 (47.65, 157.32)	4604.17 (2477.72, 6811.65)	34.91 (18.79, 51.65)	−3.50 (−3.82, −3.17)	35,020.07 (27,605.86, 44,748.24)	1789.91 (1410.96, 2287.12)	6497.93 (4601.51, 8557.33)	545.90 (386.58, 718.91)	−3.50 (−3.82, −3.17)
High-income Asia–Pacific	390.28 (307.66, 498.71)	19.95 (15.72, 25.49)	72.41 (51.28, 95.36)	6.08 (4.31, 8.01)	−4.68 (−5.04, −4.31)	475,441.27 (457,172.03, 495,269.48)	10,578.36 (10,171.88, 11,019.53)	107,205.78 (90,868.58, 123,472.32)	2666.52 (2260.16, 3071.11)	−4.68 (−5.04, −4.31)
High-income North America	5298.25 (5094.63, 5519.27)	117.88 (113.35, 122.80)	1194.73 (1012.63, 1375.95)	29.72 (25.19, 34.22)	−4.08 (−4.37, −3.79)	360,989.32 (262,784.63, 503,525.03)	2067.31 (1504.91, 2883.59)	94,943.09 (66,952.84, 123,029.41)	797.57 (562.44, 1033.51)	−4.08 (−4.37, −3.79)
North Africa and Middle East	9903.56 (5732.29, 14,710.59)	94.71 (54.82, 140.67)	4045.90 (2319.16, 6083.73)	34.22 (19.61, 51.45)	−3.47 (−3.62, −3.32)	888,609.79 (514,424.43, 1,319,945.03)	8497.55 (4919.31, 12,622.30)	363,016.79 (208,093.45, 545,949.08)	3070.17 (1759.92, 4617.29)	−3.47 (−3.62, −3.32)
Oceania	116.12 (40.84, 219.48)	53.05 (18.66, 100.27)	140.15 (64.67, 241.70)	33.99 (15.68, 58.62)	−1.75 (−2.00, −1.50)	10,418.85 (3665.41, 19,683.59)	4759.66 (1674.48, 8992.09)	12,573.56 (5804.45, 21,680.66)	3049.25 (1407.65, 5257.83)	−1.75 (−2.00, −1.50)
South Asia	27,645.81 (11,905.46, 53,727.52)	84.98 (36.59, 165.15)	7436.59 (3956.69, 11,678.71)	24.11 (12.83, 37.87)	−4.25 (−4.57, −3.94)	2,481,004.69 (1,068,360.03, 4,821,678.98)	7626.04 (3283.89, 14,820.73)	667,250.48 (355,049.10, 1,047,731.12)	2163.50 (1151.21, 3397.17)	−4.25 (−4.57, −3.94)
Southeast Asia	4614.77 (1962.90, 7686.25)	38.85 (16.53, 64.72)	1570.99 (682.50, 2349.20)	14.19 (6.16, 21.21)	−3.63 (−3.77, −3.48)	414,328.00 (176,146.01, 690,161.89)	3488.48 (1483.08, 5810.89)	141,036.47 (61,255.89, 210,893.64)	1273.62 (553.17, 1904.46)	−3.63 (−3.77, −3.48)
Southern Latin America	526.10 (392.81, 702.21)	51.05 (38.12, 68.14)	118.38 (86.64, 160.09)	15.41 (11.28, 20.84)	−3.93 (−4.61, −3.24)	47,212.70 (35,249.10, 63,019.06)	4581.50 (3420.56, 6115.34)	10,626.52 (7777.35, 14,370.07)	1383.49 (1012.55, 1870.88)	−3.93 (−4.61, −3.24)
Southern Sub-Saharan Africa	385.17 (148.32, 857.79)	24.82 (9.56, 55.27)	286.95 (107.65, 683.35)	18.03 (6.76, 42.94)	−1.03 (−1.25, −0.80)	34,562.24 (13,313.78, 76,946.58)	2226.91 (857.83, 4957.80)	25,747.13 (9660.41, 61,298.59)	1617.99 (607.07, 3852.10)	−1.03 (−1.25, −0.80)
Tropical Latin America	198.98 (165.11, 234.79)	6.14 (5.09, 7.24)	118.69 (91.55, 150.34)	3.48 (2.68, 4.40)	−1.94 (−2.58, −1.30)	17,851.94 (14,813.93, 21,065.62)	550.57 (456.88, 649.69)	10,650.49 (8214.73, 13,490.37)	311.90 (240.57, 395.07)	−1.94 (−2.58, −1.30)
Western Europe	4029.65 (3847.62, 4219.07)	88.70 (84.69, 92.87)	409.79 (343.49, 488.64)	10.04 (8.41, 11.97)	−6.73 (−7.13, −6.32)	361,551.14 (345,207.13, 378,555.97)	7958.31 (7598.55, 8332.61)	36,780.10 (30,829.46, 43,856.47)	900.90 (755.15, 1074.23)	−6.73 (−7.13, −6.32)
Western Sub-Saharan Africa	6097.46 (3040.05, 9540.61)	72.17 (35.98, 112.92)	7891.49 (3861.73, 11,892.59)	46.60 (22.80, 70.22)	−1.22 (−1.33, −1.11)	547,095.41 (272,748.98, 856,151.09)	6475.54 (3228.32, 10,133.59)	707,895.69 (346,509.41, 1,066,855.53)	4179.95 (2046.05, 6299.52)	−1.22 (−1.33, −1.11)

Abbreviations: EAPC stands for estimated annual percentage change; SDI indicates Sociodemographic Index; and UI denotes uncertainty interval.

## Data Availability

The study’s original contributions are included in the article and Supplementary Materials.

## Supplementary Materials

The following supporting information can be downloaded at: https://www.mdpi.com/article/10.3390/children12060747/s1. Figure S1: DALYs due to SIDS significantly decreased across all infant age groups from 1991 to 2021; Figure S2: DALYs changes due to SIDS across five SDI regions from 1991 to 2021; Figure S3: DALYs exhibited considerable variability in 21 regions between 1991 and 2021; Figure S4: DALYs due to SIDS varied in sexes in 2021; Figure S5: Global variation in SIDS DALYs rates among infants in different countries based on SDIs in 2021; Figure S6: National variation in SIDS DALYs rates in 2021; Table S1: Socio-demographic Index (SDI) Classification
