# Supplementary material for: Sudden Infant Death Syndrome Mortality Trends and Socioeconomic Inequalities Worldwide: Evidence from the Global Burden of Disease Study
Source: Children (Basel). 2025 Jun 9;12(6):747. doi: 10.3390/children12060747 (PMC12191546; doi:10.3390/children12060747)

Table S1. **SDI Classification.**

SDI Region	SDI Value Range	Description
Low SDI	0 - 0.4658	Lowest socioeconomic development
Low-middle SDI	0.4658 - 0.6188	Below-average socioeconomic development
Middle SDI	0.6188 - 0.7120	Average socioeconomic development
High-middle SDI	0.7120 - 0.8103	Above-average socioeconomic development
High SDI	0.8103 - 1.0000	Highest socioeconomic development

Figure S1. DALYs due to SIDS significantly decreased across all infant age groups from 1991 to 2021.

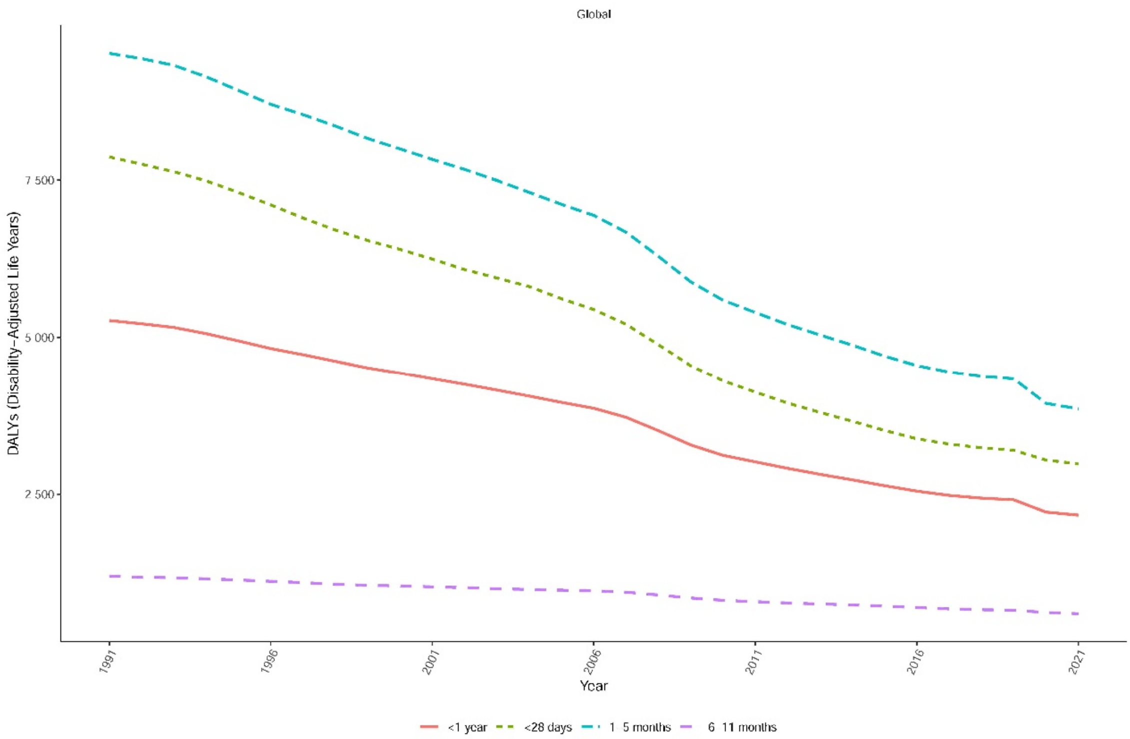

Figure S2. DALYs changes due to SIDS across five SDI regions from 1991 to 2021.

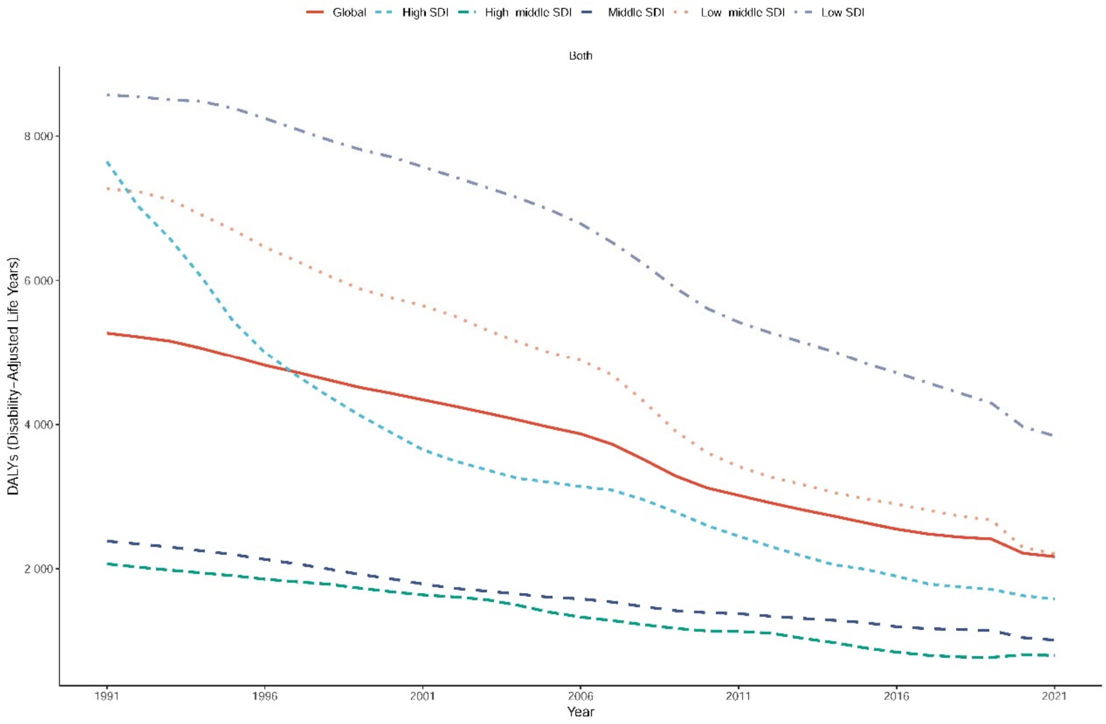

Figure S3. DALYs exhibited considerable variability in 21 regions between 1991 and 2021.

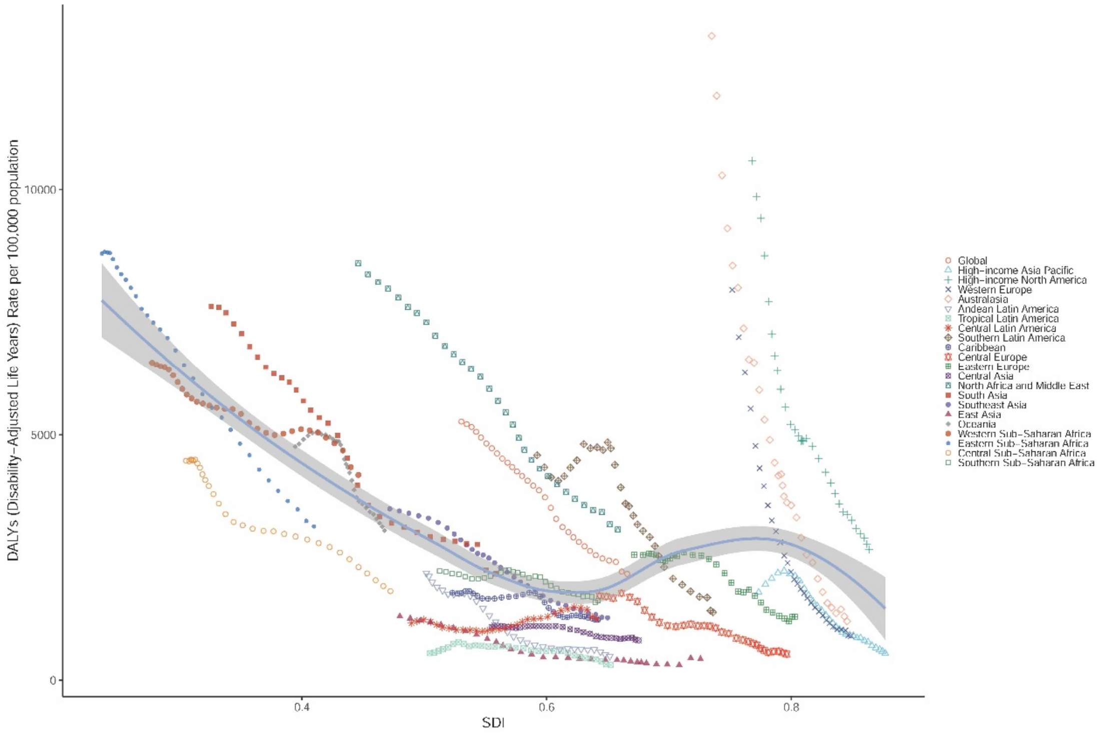

Figure S4. DALYs due to SIDS varied in sexes in 2021.

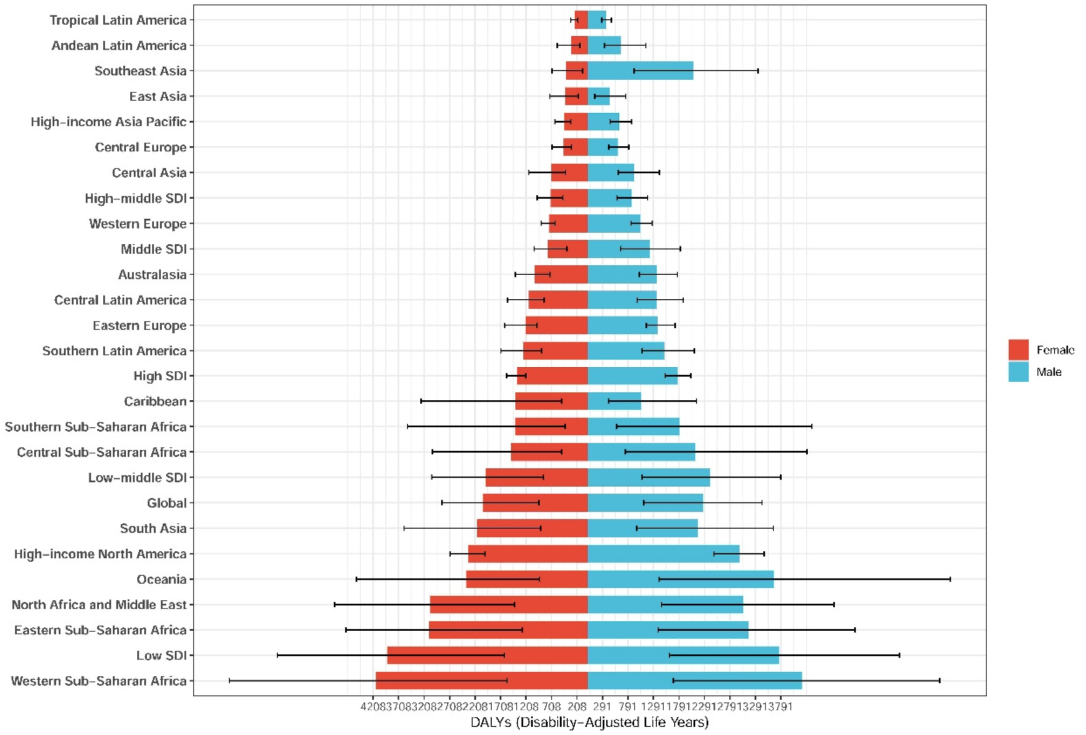

Figure S5. Global variation in SIDS DALYs rates among infants in different countries based on SDIs in 2021.

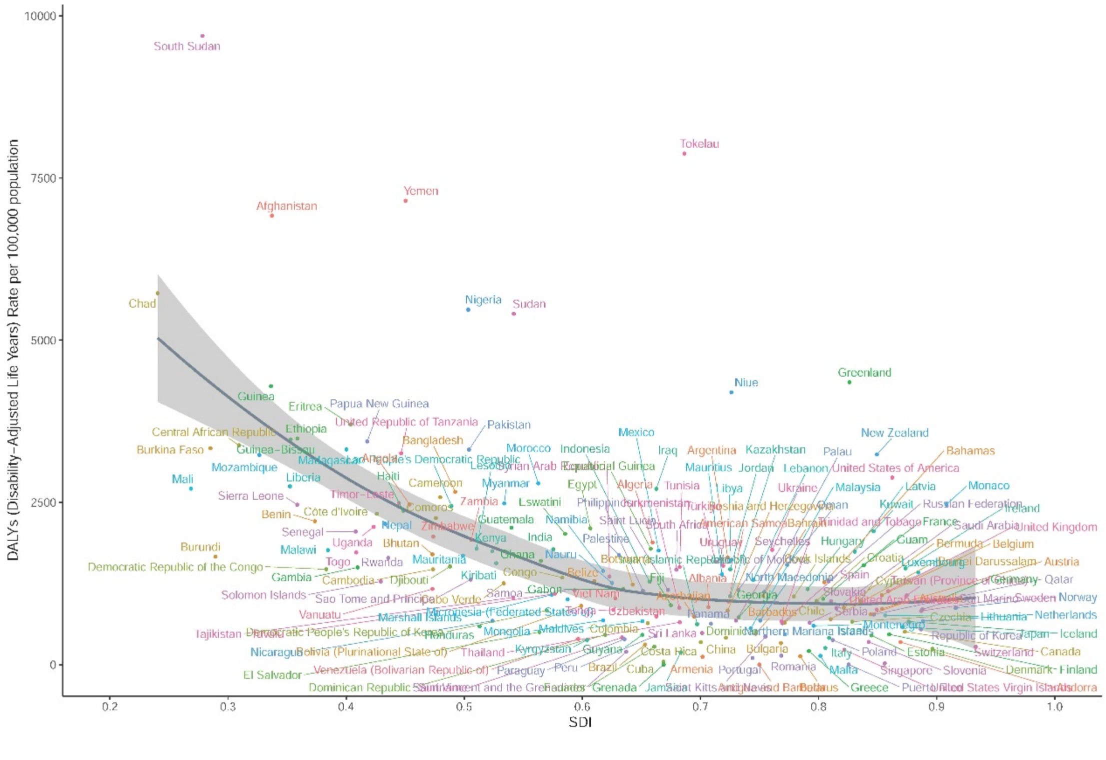

Figure S6. National variation in SIDS DALYs rates in 2021.

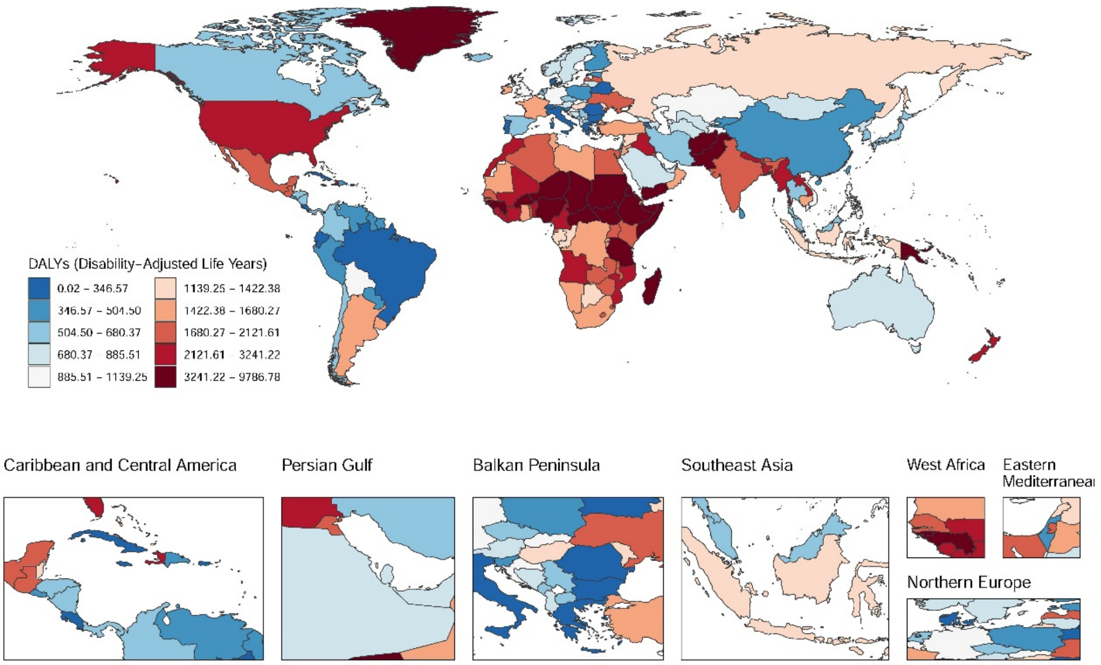

Supplement: Supplementary file 1 [file children-12-00747-s001.zip › children-3632719-supplementary.pdf]
